# The intestinal microbiome and the leaky gut as therapeutic targets in alcoholic liver disease

**DOI:** 10.3389/fphys.2012.00402

**Published:** 2012-10-11

**Authors:** Phillipp Hartmann, Wei-Chung Chen, Bernd Schnabl

**Affiliations:** ^1^Department of Medicine, University of California San DiegoLa Jolla, CA, USA; ^2^Department of Medicine, The Methodist Hospital, Weill Cornell CollegeHouston, TX, USA

**Keywords:** alcoholic liver disease, microbiome, bacterial translocation, bacterial overgrowth, bacterial dysbiosis, steatohepatitis

## Abstract

Alcoholic liver disease (ALD) encompasses hepatic steatosis, which may progress to alcoholic hepatitis, fibrosis, and cirrhosis. It remains a leading cause of morbidity and mortality in the US and worldwide. The severity of liver disease correlates with plasma levels of bacterial products in patients, and experimental ALD depends on the level of gut derived bacterial products in rodents. Since intestinal decontamination and deficiency of bacterial product receptors or their downstream signaling molecules protect from alcohol-induced liver disease, bacterial translocation (BT), qualitative, and quantitative changes of the enteric microbiome are considered as being of fundamental importance in the pathogenesis of ALD. Recent enhancements in diagnostic technologies provide a better insight into these shifts. This review highlights vital events in ALD such as BT, the importance of Toll-like receptor (TLR) signaling, intestinal bacterial overgrowth (IBO), and changes in the intestinal microbiome. Furthermore, a treatment trial section of patients reviews possible future options of therapy for ALD modifying the enteric microbiome.

## Introduction

Liver cirrhosis is the 12th leading cause of death in the United States, and 48% of all cirrhotic deaths are alcohol-related (Yoon and Yi, 2010). Hepatic steatosis or fatty liver is the most common hepatic phenotype of alcoholic liver disease (ALD) and is characterized by excessive fat accumulation in hepatocytes. It may progress to alcoholic hepatitis, fibrosis, and cirrhosis (Adachi and Brenner, 2005; Tilg and Day, 2007). Hepatocyte injury and infiltration of the liver by inflammatory cells including neutrophils are found in alcoholic hepatitis. Finally, alcoholic fibrosis is characterized by excessive accumulation of extracellular matrix proteins, most commonly in response to inflammation of the liver. Advanced liver fibrosis may lead to cirrhosis, liver failure, and portal hypertension. In many cases, liver transplantation is needed. Fatty liver can be reversed within a few weeks of abstinence (Lieber et al., 1965). However, it constitutes a risk factor for progression to fibrosis and cirrhosis in patients with severe steatosis that continue to consume alcohol (Sorensen et al., 1984; Teli et al., 1995). The risk of developing liver cirrhosis increases with elevated alcohol intake. An augmented risk was established in men with a daily alcohol intake above 60–80 g and in women above 20 g. Yet, only 6–41% of the subjects with excessive alcohol consumption develop cirrhosis (Mandayam et al., 2004). 46% of all patients with decompensated cirrhosis at the time of diagnosis die within 2 years (D'Amico et al., 2006). Mortality from ALD has been decreasing in recent years, likely due to improvements in clinical management of common complications including portal hypertension and bleeding esophageal varices (Paula et al., 2010). Nevertheless, a Danish study found 27- and 35-fold excessive mortality from alcoholic cirrhosis in men and women, respectively (Kamper-Jorgensen et al., 2004). The infection-attributed mortality in general of bacterial infections in cirrhotic patients has been reported to be 30–50% which could be caused by residing intestinal bacteria as in spontaneous bacterial peritonitis (SBP) or by non-intestinal bacteria as in respiratory tract infections or in tuberculosis with an overall similar fatality (Christou et al., 2007; Arvaniti et al., 2010). Infections in subjects with cirrhosis—all causes combined—increase mortality by 4-fold; 30% of patients succumb to bacterial infections within the first month and another 30% die by 1 year (Arvaniti et al., 2010).

There is strong evidence for a gut-liver axis that is causatively linked not only to progression of alcohol-induced liver disease but also to infections, both in patients and experimental animal models.

This review highlights central events in ALD such as bacterial translocation (BT), Toll-like receptor (TLR) stimulation via bacterial ligands and consequent steps, intestinal bacterial overgrowth (IBO), and dysbiosis, as well as clinical trials of various treatment modalities of patients with ALD. It additionally refers to other etiologies of liver disease and the implication of the microbiome/bacteria therein.

## Bacterial translocation

BT is defined as migration of viable bacteria or bacterial products from the gastrointestinal tract through the epithelium to mesenteric lymph nodes or other extraintestinal organs (Berg and Garlington, 1979). BT is initiated when there is damage to the epithelium (Mathurin et al., 2000; Parlesak et al., 2000; Keshavarzian et al., 2001; Rao et al., 2004; Purohit et al., 2008). In Caco-2 cell layers and intestinal biopsies, it has been demonstrated that acetaldehyde, an indirect oxidized metabolite of alcohol, disrupts the intestinal barrier (Rao, 1998; Basuroy et al., 2005; Purohit et al., 2008). Ethanol administration results in acute damage of the colonic epithelial barrier through acetaldehyde (metabolized by the intestinal flora) in rats and subsequently activates mast cells (Ferrier et al., 2006). In humans, ethanol intake leads to duodenal and jejunal mucosal injury (Bode and Bode, 2003). Lipopolysaccharide (LPS), a critical component of the outer membrane of Gram-negative bacteria (Fadl et al., 2005), induces macrophages to release pro-inflammatory cytokines resulting in liver injury (French, 2001). Pro-inflammatory cytokines such as IL-1β and tumor necrosis factor (TNF) have been shown to be elevated in the distal ileum of mice fed with ethanol for 2 weeks (Fleming et al., 2001). They are also known to disrupt the intestinal barrier (Wang et al., 2005; Clayburgh et al., 2006; Al-Sadi et al., 2008). This impairment has been demonstrated in duodenal biopsies of cirrhotic patients, which shows enlarged intercellular spaces below the tight junctions (Such et al., 2002). In another study, the augmented intestinal permeability existed longer than 14 days after having eschewed drinking ethanol (Bjarnason et al., 1984). In summary, these findings indicate that morphologic and functional changes may persist in the gut barrier after long-term alcohol abuse similar to lasting structural and functional alterations in the liver.

To assess the quantity of BT, bacterial products (also called pathogen-associated molecular patterns or PAMPs) or viable bacteria can be measured in the portal or systemic circulation. Plasma LPS levels were elevated in patients with alcoholic steatohepatitis and alcoholic cirrhosis (Bode et al., 1987; Fukui et al., 1991; Schafer et al., 2002). There is a direct association between ethanol administration and increased plasma LPS in animal models (Nanji et al., 1993; Adachi et al., 1995; Tamai et al., 2000). Equally in patients, endotoxemia is present in early stage of ALD prior to the onset of fibrosis or cirrhosis (Parlesak et al., 2000; Bode and Bode, 2005). The severity of liver injury positively correlates with plasma LPS levels in patients with cirrhosis (Lin et al., 1995), which could also be related to a decreased clearance of endotoxin from the blood by the liver (Satoh et al., 2008). Interestingly, plasma LPS is higher in subjects with alcoholic cirrhosis than in subjects with cirrhosis from other causes (Bode et al., 1987; Fukui et al., 1991). Peptidoglycan, the major component of Gram-positive bacterial cell walls, is higher in rat plasma following acute ethanol feeding (Tabata et al., 2002). Bacterial DNA, a surrogate marker of BT, is higher in cirrhotic rats (Guarner et al., 2006). BT even precedes IBO and intestinal dysbiosis in ALD (Yan et al., 2011) similarly to CCl_4_-induced liver injury (Fouts et al., 2012).

The innate immune system has conserved pattern recognition receptors, e.g., TLRs, that recognize specific PAMPs such as LPS, peptidoglycan, lipoproteins, lipoteichoic acid, double-stranded RNA, and unmethylated DNA (Akira et al., 2006). TLR2 is important in detecting Gram-positive bacteria and recognizes a number of bacterial components such as lipoproteins, peptidoglycan, and lipoteichoic acid (Aliprantis et al., 1999; Schwandner et al., 1999; Yoshimura et al., 1999). According to one study, cirrhotic patients with ascites had an increased risk of developing SBP in the presence of a specific TLR2 genotype (16934 TT genotype) or a TLR2 GT microsatellite polymorphism; presence of both risk factors further augmented the susceptibility for SBP (Nischalke et al., 2011).

The cellular receptor for endotoxin is TLR4, which plays a central role in the innate immune response to BT. After binding of LPS to its cellular receptor TLR4, MyD88-dependent and MyD88-independent (TRIF/IRF-3-dependent) pathways are activated. TLR4 mutant C3H/Hej mice and TLR4 knock-out mice had mitigated hepatic steatosis, inflammation and necrosis in ALD compared to wild type mice (Uesugi et al., 2001; Hritz et al., 2008). TLR4 signaling is important in both BM-derived cells including Kupffer cells, and endogenous liver cells including hepatic stellate cells (HSCs) for alcohol-induced hepatocyte injury, steatosis, inflammation, and fibrogenesis (Inokuchi et al., 2011). Activation of the MyD88- independent, TRIF/IRF-3-dependent pathway in the liver plays a major role in experimental ALD (Hritz et al., 2008; Petrasek et al., 2011). A comparable significance of TLR4 for liver fibrogenesis has been noted in cholestatic or toxic liver fibrosis in mice (Seki et al., 2007). Other reports indicate a similar implication of TLR4 in non-alcoholic steatohepatitis in rodents (Ye et al., 2012). The implication of TLR4 in liver disease has also been proven in human subjects: it is one of seven genes linked to an elevated risk of developing cirrhosis in patients with chronic hepatitis C (Huang et al., 2007). Specific single nucleotide polymorphisms (TLR D299G and T399I) are associated with less hepatic fibrosis by decreased TLR4-mediated signaling (Guo et al., 2009). Similarly, mice deficient in TLR9, a recognition receptor activated by CpG motifs specific to bacterial DNA, are protected from hepatic fibrosis (Gabele et al., 2008). Deficiency in cluster of differentiation 14 (CD14) as cellular co-receptor for LPS protects from alcohol-induced liver injury (Yin et al., 2001). Similarly, CD14 knockout mice are resistant to liver injury from experimental cholestasis and NASH (Isayama et al., 2006; Imajo et al., 2011). Lastly, selective intestinal decontamination with antibiotics results in a decline in plasma LPS and consequently, this results in an attenuated liver damage in preclinical animal models of ALD (Adachi et al., 1995; Enomoto et al., 1999, 2001).

Following LPS binding to TLR4 and the initiation of intracellular downstream signaling, a complex interplay occurs between innate immune cells and other cells of the body via the induction of cytokines and chemokines. It comprises the migration of inflammatory cells into the liver, such as neutrophils, leukocytes, monocytes, and macrophages. Recruited hepatic macrophages and resident Kupffer cells play a key role in the pathogenesis of ALD because inactivation of Kupffer cells through gadolinium chloride injections largely alleviates alcohol-induced liver disease (Adachi et al., 1994; Koop et al., 1997). During hepatic inflammation, Kupffer cells produce a large variety of cytokines such as TNF-α, IL-6, IL-1, chemokines such as KC (CXCL1), MIP-2 (CXCL2), MCP-1 (CCL2), MIP-1α (CCL3), MIP-1β (CCL4), and RANTES (CCL5), and reactive oxygen species (Seki and Schnabl, 2012). In addition, they also produce pro-fibrogenic mediators, such as TGF-β and PDGF which activate quiescent HSCs to produce extracellular matrix proteins (Bataller and Brenner, 2005). Oxidative stress via alcohol and acetaldehyde sensitizes HSC to activation by LPS and consequent induction of hepatic fibrosis (Quiroz et al., 2001; Karaa et al., 2008). Similarly to Kupffer cells, HSC upon LPS stimulation express pro-inflammatory cytokines (e.g., TNF-α, IL-6) and chemokines such as CXCL1 (Schwabe et al., 2003; Seki et al., 2007). In hepatocytes, LPS can promote apoptosis, especially in conjunction with other hepatotoxic agents (Nagaki et al., 1999; Kudo et al., 2009). Thus, endotoxin affects Kupffer cells, hepatocytes, and HSC to participate in the initiation and progression of ALD.

The detailed role of the adaptive immune system for the initiation and progression of ALD remains largely unknown (Gao and Bataller, 2011).

Thus, BT not only contributes to increased infection rates and mortality in end-stage liver disease but also to disease progression in early stages of alcoholic liver injury and disease. Viable bacteria need to translocate from the intestine to extraintestinal sites to cause infections, while translocation of bacterial products is sufficient to cause disease progression in ALD.

## Intestinal bacterial overgrowth

### Overgrowth of intestinal bacteria in experimental ALD

The human intestine harbors 10^13^ bacteria, a quantity that outnumbers the total sum of cells in the human body of 10^12^ (Guarner and Malagelada, 2003; O'Keefe, 2008). The impressive density of 10^3^ microbial cells/g contents in the jejunum mounts up to 10 ^11^cells/g contents in the colon (Savage, 1977). Mice fed with alcohol via an intragastric feeding tube for 3 weeks show IBO (Yan et al., 2011). Both aerobic and anaerobic bacteria increase predominantly in the small intestine (Yan et al., 2011). IBO correlates well with increased BT. For example, experimentally induced IBO leads to BT and subsequent liver injury (Lichtman et al., 1990). On the other hand, selective intestinal decontamination decreases BT and plasma LPS, and ameliorates the detrimental effect of LPS on the liver (Adachi et al., 1995; Enomoto et al., 1999, 2001). Bacterial overgrowth and BT are also common characteristics observed in end-stage liver disease (Parks et al., 1996; Guarner et al., 1997). Cirrhotic rats with BT have been shown with increased bacterial overgrowth (Runyon et al., 1994; Garcia-Tsao et al., 1995; Sanchez et al., 2005). Taken together, bacterial overgrowth in the intestine occurs already in an early stage of experimental ALD. In addition to alcohol, other experimental etiologies of liver disease, such as cholestasis, toxic, and non-alcoholic fatty liver disease, are accompanied by IBO (Wigg et al., 2001; Miele et al., 2009; Fouts et al., 2012).

### Intestinal bacterial overgrowth in patients with ALD

There are different diagnostic tests to detect IBO in patients. Aside from D-Xylose test where D-xylose is ingested and then measured in the urine and blood (Craig and Atkinson, 1988; Craig and Ehrenpreis, 1999), there are other breath tests used in the diagnosis of IBO. Hydrogen breath tests are based on the fact that the only source for hydrogen gas in human intestine is bacterial metabolism of carbohydrates (Levitt, 1969). For these tests, carbohydrates are ingested and the intestinal bacteria metabolize carbohydrates into hydrogen that will be detected in the breath. The most frequently used substrates for diagnosis of IBO are glucose (Metz et al., 1976; Kerlin and Wong, 1988) and lactulose (Rhodes et al., 1979; Simren and Stotzer, 2006). Tests with labeled carbon take advantage of the bacteria's ability to deconjugate bile acids. For example in the glycocholic acid breath test, ^14^C glycocholic acid is administered and the ^14^CO_2_ is then measured (Donald et al., 1992). However, the gold standard to prove small-intestinal bacterial overgrowth (SIBO) is culturing of jejunal aspirates of bacteria to demonstrate at least 10^5^ colony forming units/ml (Kerlin and Wong, 1988; Bauer et al., 2000; Simren and Stotzer, 2006).

Similar to findings in animal models, human subjects with SIBO exhibit an increased intestinal permeability (Riordan et al., 1997; Miele et al., 2009). It is now well-established that IBO occurs more commonly in patients with ALD. Aerobic and anaerobic bacteria are increased in jejunal aspirates from subjects with long-term alcohol use (Bode et al., 1984; Casafont Morencos et al., 1996). SIBO is more frequent in cirrhotic patients and directly correlates with the severity of liver damage (Casafont Morencos et al., 1996; Bauer et al., 2001; Pande et al., 2009; Jun et al., 2010). Equally, a higher prevalence of antibodies directed against bacterial products is noted in more advanced liver disease (Papp et al., 2010).

Several hypotheses have been raised to explain the pathogenesis of bacterial overgrowth: impaired bile flow, intestinal dysmotility, less acidic gastric pH, and altered intestinal innate immune response.

#### Impaired bile flow

In experimental mouse models, the antimicrobials angiogenin 1 and RNAse family member 4 were found to be targets of the nuclear receptor FXR in intestinal epithelial cells, the receptor for bile acids. A decrease in these antimicrobials is associated with IBO (Inagaki et al., 2006). Oral feeding of bile acids to rats with cirrhosis has been shown to reverse bacterial overgrowth in the intestine and to lower BT and plasma LPS levels (Lorenzo-Zuniga et al., 2003). Hence, the markedly decreased bile acid secretion in cirrhotic patients into the intestine (Raedsch et al., 1983) may contribute to IBO.

#### Intestinal dysmotility

Ethanol decreases intestinal motility which may in turn cause luminal bacteria to proliferate (Bode and Bode, 2003). Patients with cirrhosis tend to have prolonged orocecal transit time (Madrid et al., 1997; Chang et al., 1998; Gunnarsdottir et al., 2003). Similarly, SIBO with a delayed transit time was noted more commonly in cirrhotic subjects with hepatic encephalopathy (Gupta et al., 2010). On the other hand, cisapride, a pro-motility drug, increases small intestinal motility and thereby suppresses bacterial proliferation in cirrhotic patients (Madrid et al., 2001).

#### Altered gastric pH

Hypochlorhydria has been linked to IBO in the jejunum of subjects with cirrhosis (Shindo et al., 1993; Bauer et al., 2001). Use of proton pump inhibitors in patients with cirrhosis increases the risk of suffering SBP (Bajaj et al., 2009). Thus, impaired bile flow, intestinal dysmotility and less acidic gastric pH, either separately or in a more intertwined fashion, promote bacterial overgrowth and further leads to development of ALD.

#### Altered intestinal innate immune response

As stated earlier, host antimicrobial molecules are an integral part of the intestinal innate immune system that regulates the total bacterial burden, distribution, and composition in the intestine. These bactericidal proteins are secreted from Paneth cells and intestinal epithelial cells. Regenerating islet derived (Reg)-3b and Reg3g, two of the bactericidal proteins, have been found to be suppressed in murine and human small intestine after ethanol feeding and chronic alcohol abuse, respectively (Yan et al., 2011). Administration of prebiotics has been shown to restore Reg3g protein levels, and this helps to reverse bacterial overgrowth and to ameliorate experimental alcoholic steatohepatitis.

Thus, quantitative changes in the intestinal microflora predispose to BT and can be strongly considered a contributing factor to the progression of ALD.

## Enteric microbiome

### Intestinal dysbiosis in experimental ALD

The intestine provides residence to a variety of microbial communities consisting of 10 different bacterial phyla with more than 15,000 species-level bacterial phylotypes (Camp et al., 2009). The most abundant of these phylotypes in mice and humans are Firmicutes and Bacteroidetes (Eckburg et al., 2005; Ley et al., 2006; Yan et al., 2011). In a healthy state, the intestinal microflora keeps a symbiotic relationship with its host. The total bacterial burden, distribution, and composition are regulated by the host's immune system via intestinal antimicrobial proteins. Dysbiosis is known as an imbalance between enteric microbial colonies and its associated deleterious effects on the colonized host (McLoughlin and Mills, 2011), and this has been implicated in diseases including inflammatory bowel disease (Frank et al., 2007). Only a minority of intestinal bacteria can be cultured by conventional culture techniques, but rapid advances in analytic methods have intensely enlarged our ability to study biodiversity in a microbial community (Eckburg et al., 2005; Gill et al., 2006). Offering a first glimpse into alcoholic enteric dysbiosis by using Length Heterogeneity PCR (LH-PCR) fingerprinting, one study noted a significantly changed intestinal microbiome in rats after 10 weeks of ethanol administration (Mutlu et al., 2009). It reports that probiotic or prebiotic treatment protected alcohol fed rodents from intestinal dysbiosis. Another study that performed deep DNA pyrosequencing of bacterial 16S rRNA after 3 weeks of continuous intragastric ethanol administration showed significant intestinal dysbiosis in alcohol fed mice. Dysbiosis was characterized by a profound suppression of several endogenous probiotic bacteria such as *Lactobacillus* (Yan et al., 2011). This is especially noteworthy as several studies have demonstrated a beneficial effect of *Lactobacillus* supplementation in experimental ALD (Nanji et al., 1994; Forsyth et al., 2009; Mutlu et al., 2009). The question arises as to whether an alcohol-associated microbiome in mice is specific for alcohol or similar changes can be also observed in other experimental liver disease models. In cholestasis, 16S rRNA sequencing could not show a significant qualitative change in the microbiome relative to control mice. In mice with CCl_4_-induced toxic liver injury, dysbiosis could be observed with an elevated intestinal burden of Firmicutes and Actinobacteria (Fouts et al., 2012). In contrast to microbial changes in ALD, obesity-induced fatty liver seems to be associated with an augmented bacterial load of Firmicutes and a relative decrease in Bacteroidetes (Ley et al., 2005; Turnbaugh et al., 2009). Although experimental liver disease models depend on translocated bacterial products from the intestine, there are distinct changes in the enteric microbiome of four different experimental etiologies of liver disease.

### Intestinal dysbiosis in patients with ALD

As mentioned above, the two most abundant bacterial phyla in the intestine of humans are Firmicutes and Bacteroidetes (Eckburg et al., 2005; Ley et al., 2006). According to one study, Bacteroidetes were found to be significantly reduced and Proteobacteria and Fusobacteria highly enriched in patients with cirrhosis related to hepatitis B and alcohol abuse (Chen et al., 2011). Specifically, the families Lachnospiraceae (Chen et al., 2011) and Ruminococcaceae were decreased in patients with cirrhosis, whereas Enterobacteriaceae, Alcaligeneceae, and Fusobacteriaceae were significantly higher (Bajaj et al., 2012). In another study, a significant decrease in various *Lactobacilli* was demonstrated in human subjects with hepatitis B virus-related decompensated cirrhosis or liver transplant for hepatitis B cirrhosis (Wu et al., 2011). This might potentially explain why administration of *Lactobacillus* is beneficial in certain types of liver injury (Kirpich et al., 2008; Vajro et al., 2011). Recently, Bacteroidacea from the phylum Bacteroidetes have been found to be decreased in patients with chronic alcohol abuse compared to healthy controls (Mutlu et al., 2012). Compared to non-dysbiotic subjects, alcoholics with dysbiosis had smaller intestinal quantities of Bacteroidetes and higher levels of Proteobacteria. Contrary to the findings in experimental ALD in mice (Yan et al., 2011), the two aforementioned studies demonstrated a reduction of intestinal Bacteroidetes in patients with (at least partly) alcohol-induced cirrhosis (Chen et al., 2011; Mutlu et al., 2012). This difference might be due to a later stage of liver disease in patients and increased heterogeneity of patient population in these human studies as compared to well-controlled animal studies. In addition, different methods for sample collection, storage, DNA extraction, and sequencing might also explain these differences.

Many of the translational human microbiome studies are descriptive in nature. To advance the field and link changes in the microbiome to onset and progression of disease, further metagenomic, transcriptomic, and in particular metabolomic studies are urgently needed. It is conceivable that not only translocation of PAMPs contributes to ALD, but changes in bacterial metabolites further modify liver disease.

### The microbiome as target in alcoholic liver disease

An intricate interaction exists between bowel flora and liver in ALD. Alteration of normal bowel flora and significant overgrowth of harmful bacteria are described in animal models and humans with liver disease. These bacteria release endotoxins that increase damage to the integrity of gut flora and activate certain inflammatory pathways that lead to progression of alcohol liver disease (Forsyth et al., 2009). High endotoxin environment stimulates secretion of cytokines such as TNF-α, IL-1, and IL-6 that influence the development of liver fibrosis and cirrhosis (Gratz et al., 2010). Gut bacteria play a major role in the pathogenesis of ALD, and there are human trial studies that have shown that antibiotics and probiotics are effective in reducing the number of gram-negative bacteria and altering the gut flora to prevent further alcohol-induced liver injury and liver fibrosis. In addition, studies have demonstrated in human trials that use of probiotics can improve liver function by decreasing oxidative damage/stress, improving neutrophil function, and reducing endotoxin levels.

#### Antibiotics

Antibiotics have shown to provide beneficial effects in animal models with liver disease. Prophylactic use of antibiotics in patients with chronic liver disease is an established method of preventing infections in upper gastrointestinal hemorrhage and recurrent SBP (Leber et al., 2012). A randomized controlled trial showed that long-term prophylactic use of ciprofloxacin reduced the 1 year mortality rate in cirrhotic patients with low ascitic protein levels and without prior SBP episode (Terg et al., 2008). Similarly, a double blind, multi-center, placebo-controlled study investigating the long-term efficacy of norfloxacin in cirrhotic patients who had survived a previous episode of SBP found a significant reduction of the risk of SBP recurrence in the treated group at 1 year of follow-up (Gines et al., 1990).

Despite the established role of antibiotics in patients with hepatic encephalopathy, there are few human trials that have demonstrated the positive role of antibiotics in patients with ALD. According to one randomized controlled trial by Madrid et al., nine patients with alcoholic cirrhosis were treated with antibiotics that consisted of norfloxacin and neomycin during a period of 6 months. There was improvement of Child-Pugh status at 3 and 6 months treatment period (Madrid et al., 2001). Thus, long-term antibiotic treatment may improve the prognosis of ALD and result in a higher survival of cirrhotic patients. Despite improvement of liver function and reduction of infections in patients with ALD, it is also important to consider the negative effects of antibiotics on the gut mucosa. Prolonged use of antibiotics can sometimes increase the vulnerability of gut flora and lead to increased pathogenic bacterial colonization (Brandl et al., 2008). In addition, it is also important to consider that continued use of antibiotics can result in heightened bacterial resistance to these antibiotics and increased virulence of these pathogens (Novella et al., 1997; Campillo et al., 1998; Fernandez et al., 2002).

#### Probiotics

Probiotics are living non-pathogenic microorganisms that cause the growth of other microorganisms. They possess beneficial effects on the host by changing the gut microbiota profile, and this further leads to changes in the gut lumen that promotes anti-inflammatory effects. This improves gut barrier integrity and decreases the release of pro-inflammatory products by harmful bacteria (Forsyth et al., 2009; Gratz et al., 2010). By enhancing production of anti-inflammatory cytokines and secretion of antibacterial proteins, this further helps to reduce the production and translocation of bacterial endotoxin (Bongaerts et al., 2005; Forsyth et al., 2009; Gratz et al., 2010; Wang et al., 2011). Probiotics include lactic acid bacteria such as *Lactobacilli*, *Lactococci*, and *Bifidobacteria*, or yeasts such as *Saccharomyces cerevisiae* (Bongaerts et al., 2005). Probiotics have been shown in several human clinical trials to prevent recurrent *Clostridium difficile* colitis and to maintain remission of pouchitis (Kirpich et al., 2008).

Probiotics including *Lactobacillus* and *Bifidobacterium* species have been used in human trials to study the beneficial effects of these microorganisms in patients with ALD. An investigation by Kirpich et al. consisted of a randomized, prospective study of 66 alcoholic male patients and 24 healthy, adult male controls concerning the therapeutic role of probiotics in treatment of ALD (Kirpich et al., 2008). In this study, alcoholic patients had reduced numbers of *Lactobacilli* and *Enterococci*, and increased number of *E. coli*. Of the alcoholic patients that received probiotics, the depressed numbers of *Bifidobacteria*, *Lactobacilli*, and *Enterococci* returned to the levels seen in healthy controls. Initial liver function tests, specifically AST, ALT, and GGT, were significantly elevated in the alcoholic group. After 5 days of probiotic therapy, patients treated with probiotics had significantly lower AST and ALT activity compared to the control group. In the subgroup of patients with well-defined alcoholic hepatitis, there was a significant reduction in ALT, AST, GGT, LDH, and total bilirubin values after probiotic therapy. The study further emphasizes that probiotic therapy is associated with greater improvement in liver enzymes.

Probiotic treatment can also help to restore neutrophil functions in patients with alcoholic cirrhosis. In an open-label study by Stadlbauer et al., 12 patients with alcoholic cirrhosis received *Lactobacillus casei Shirota* three times daily for 4 weeks (Stadlbauer et al., 2008). This was compared to 13 healthy controls and eight cirrhotic patients that did not receive probiotics. At baseline, the study showed that alcoholic cirrhotic patients have reduced neutrophil capacity. However, 28 days of treatment with *L. casei Shirota* revealed normalization of phagocytic activity in patients with alcoholic cirrhosis in the treatment group as opposed to the control group. The study also demonstrated that it is safe to administer the probiotics in this group of patients without adverse events. Another study by Loguercio et al. evaluated the effects of chronic therapy with probiotics on patients with alcoholic liver cirrhosis (Loguercio et al., 2005). In this study, 20 patients with alcoholic liver cirrhosis were treated with the probiotic VSL#3, which is a mixture of 450 billion bacteria in various strains including *Streptococcus thermophilus*, *Bifidobacterium breve*, *B. longum*, *B. infantis*, *L. acidophilus*, *L. plantarum*, *L. casei*, and *L. bulgaricua* for 3 months. Patients treated with probiotic therapy presented with significantly reduced plasma levels of oxidative stress parameters, and there was improvement of liver function. An improvement of cytokine levels following treatment of VSL#3 was also noticed. A double-blind placebo-controlled study by Lata et al. investigated the effect of the probiotic *E. coli Nissle* on 34 patients with alcoholic cirrhosis (Lata et al., 2007). Overall, there was significant improvement of the intestinal colonization in the group treated with probiotics. In addition, an improvement of Child-Pugh Score in alcoholic cirrhotic patients treated with *E. coli Nissle* was observed. Probiotic treatment may improve the prognosis of ALD, but the effects of probiotics are strain-dependent. Therefore, further clinical studies are warranted to determine which probiotic strain should be used and which patient population should be treated.

#### Prebiotics

Prebiotics are complex carbohydrates that cannot be degraded by pancreatic and intestinal enzymes in the gastrointestinal tract (Yan et al., 2011). These carbohydrates are ultimately metabolized by gut microflora. Some examples of prebiotics include: lactulose, fructo-oligosaccharides (FOS), oats, and galacto-oligo-saccharides (Gibson, 2008; Yan et al., 2011). There is a lack of current human trials that further elucidate the beneficial effect of prebiotics on patients with ALD. However, as mentioned above, there are animal studies that have shown that prebiotics help to slow liver damage progression. Prebiotics potentially possess beneficial effects in alcohol liver disease by altering the gut microflora. However, additional clinical studies are needed to further clarify the possible benefits of the use of prebiotic therapy in patients with ALD.

#### Synbiotics

Synbiotics are mixtures of pre- and pro-biotics. Rats fed with synbiotics (*L. acidophilus, L. helveticus*, and *Bifidobacterium* in an enriched medium) displayed significantly diminished endotoxemia, BT, and liver damage in the course of acute pancreatitis and simultaneous heavy alcohol consumption (Marotta et al., 2005). A 7 day patient trial revealed a significant amelioration of Indocyanine Green clearance as measures of liver function in cirrhotic patients treated with synbiotics (*Pediacoccus pentoseceus*, *Leuconostoc mesenteroides*, *L. paracasei subspecies paracasei*, and *L. plantarum* along with bioactive, fermentable fiber: betaglucan, inulin, pectin, and starch) as opposed to the placebo-controlled group in which no change was observed (Riordan et al., 2007). This synbiotic treatment may also result in an improvement of the Child-Pugh classification with a significant improvement in serum bilirubin, albumin concentrations, and international normalized ratio (INR). Furthermore, administration of the same synbiotics to cirrhotic patients with minimal hepatic encephalopathy (MHE) leads to significantly decreased ammonia levels and a reversal of MHE in 50% of the cases (Liu et al., 2004). It was also associated with significantly lower LPS levels in the blood, as was feeding of fermentable fiber alone. A meta-analysis demonstrated that synbiotics and both pre- and pro-biotics were associated with improvement of liver cirrhosis-associated MHE (Shukla et al., 2011). Amongst the prebiotics, lactulose seems to be the agent of choice for treatment of cirrhosis-associated MHE. Rishi et al. compared treatment with synbiotics (*L. acidophilus* and inulin) to *L. acidophilus* (probiotic) and inulin (prebiotic) alone, respectively, in *Salmonella typhimurium*-induced liver injury (Rishi et al., 2009). Mice fed with synbiotics were effectively protected against BT, lipid peroxidation, and liver damage, as were pre- and pro-biotic treated groups. Nevertheless, none of the observations suggested a synergistic effect in the synbiotic-supplemented group in this model. Thus, synbiotics—despite a wide range of composition—seem to alleviate liver damage and improve the prognosis in patients with cirrhosis.

## Conclusion

ALD remains a leading cause of morbidity and mortality in the United States and worldwide. Since progression of ALD is partly driven by inflammatory responses to bacteria and their products, BT is a central event in the pathogenesis of ALD. This review highlights several mechanisms which facilitate BT, i.e., impaired bile flow, intestinal dysmotility, altered gastric pH, impaired intestinal immunity, and oxidative stress at the intestinal mucosa that increases intestinal permeability. Translocated bacterial PAMPs, in particular LPS, leads to TLR signaling and secretion of pro-inflammatory cytokines and chemokines, and this ultimately enhances alcoholic liver injury and steatohepatitis. Linked to BT and crucial in ALD are quantitative changes in the intestinal microflora. IBO is a driving force in the etiology of ALD. Given a leaky gut, overgrowth leads to increased luminal burden of bacterial products and toxins that can now traverse the gut barrier and encounter the liver as first organ in the body. This can be considered a second hit for hepatocytes that already encounter and metabolize ethanol. Recent advances in diagnostic technologies such as deep pyrosequencing have enabled a meticulous investigation of qualitative changes of the enteric microbiome. By means of these tools, dysbiosis has been described in preclinical and clinical ALD. Further studies are necessary to elucidate more clearly the exact impact of qualitative changes in the enteric flora on disease progression. Using metagenomics, transcriptomics, and metabolomics, future investigations need to focus on the identification of metabolites that mediate the effect of orally ingested alcohol to extraintestinal organs. Although there is promising but limited evidence that modulation of the microbiome using—biotics mitigate disease activity in patients, additional clinical studies on perhaps novel targets are still required before routine use of these agents is advisable.

### Conflict of interest statement

The authors declare that the research was conducted in the absence of any commercial or financial relationships that could be construed as a potential conflict of interest.

## Abbreviations

ALD: alcoholic liver disease
BT: bacterial translocation
FXR: farnesoid X receptor
IBO: intestinal bacterial overgrowth
IL: interleukin
LPS: lipopolysaccharide
MHE: minimal hepatic encephalopathy
PAMPs: pathogen-associated molecular patterns
Reg3: regenerating islet-derived 3
SIBO: small intestinal bacterial overgrowth
TLR: Toll-like receptor
TNF: tumor necrosis factor.
